# A novel surgical technique for dissection of Siewert type II adenocarcinoma of the esophagogastric junction: the transthoracic single-port assisted laparoscopic esophagogastrostomy

**DOI:** 10.1093/gastro/goab057

**Published:** 2022-04-25

**Authors:** Wenjun Xiong, Xiaohua Zhong, Yan Chen, Sijing Luo, Yaohui Peng, Yuanfa Hou, Jin Li, Yansheng Zheng, Lijie Luo, Ziming Cui, Wei Wang

**Affiliations:** 1 Department of Gastrointestinal Surgery, Guangdong Provincial Hospital of Chinese Medicine, The Second Affiliated Hospital of Guangzhou University of Chinese Medicine, 510120 Guangzhou, China; 2 Department of Gastrointestinal Surgery, Huizhou Municipal Central Hospital, Huizhou, China; 3 The Second Clinical Medical College, Guangzhou University of Chinese Medicine, Guangzhou, China; 4 General Surgery Department, Yunfu People's Hospital, Yunfu, China

## Introduction

The surgical approach for Siewert type II adenocarcinoma of the esophagogastric junction (AEG) is controversial. The JCOG9502 trial [1, 2] has shown that the left thoracoabdominal approach has not improved the prognosis but increased the morbidity in patients with Siewert type II or III AEG compared with the abdominal-transhiatal (TH) approach. However, lower mediastinum lymphadenectomy and digestive tract reconstruction using the laparoscopic TH approach are technically difficult [3]. Therefore, our center proposed transthoracic single-port assisted laparoscopic esophagogastrectomy for Siewert type II AEG. This technique makes lower mediastinum lymphadenectomy easy and efficient as well as reconstruction by opening the left diaphragm and adding the thoracic auxiliary operative port in the 6∼7 intercostal space of the anterior axillary line. Herein, we describe the surgical technique.

## Surgical procedures

### Position and port arrangement

Under general anesthesia with endotracheal intubation, the patient was maintained in the supine position with legs apart. Five ports were used: a 10-mm subumbilical port, through which the laparoscope was introduced; a 12-mm left port and a 5-mm right port along the anterior axillary line below the costal margin; and 5-mm left and right ports along the midclavicular line at the level of the umbilicus.

### Perigastric lymph nodes dissection

A diagnostic laparoscopy was conducted to exclude distant metastasis. The liver was retracted to expose the esophageal hiatus. Perigastric lymph nodes (LNs) dissection proceeded as follows. First, the gastrocolic and splenogastric ligaments were divided along the border of the transverse colon toward the spleen. The left gastroepiploic artery and short gastric arteries were ligated and divided with No. 4sa and No. 4sb LNs dissection. After the exposure of the gastropancreatic fold, the No. 11p and No. 11d LNs along the splenic vessels were removed. The posterior gastric artery that originated from the splenic artery was severed. Then the left part of the cardia was mobilized with No.2 LNs dissection. After dividing the great omentum toward the hepatic flexure of the colon, the right gastroepiploic vein and artery were ligated at the root and the No. 4d and No. 6 LNs were dissected. Subsequently, the duodenum was transected after the dissection of the No. 5 LNs. The suprapancreatic area, including the No. 7, No. 8a, and No. 9 LNs, was dissected after the left gastric artery and vein were divided. The No. 12a LNs were dissected using the portal vein approach in our previous description [4]. Finally, the No. 1 and No. 3 LNs were removed along the lesser curvature of the stomach. The signal for the completion of the perigastric lymphadenectomy was the exposure of the abdominal esophagus and diaphragmatic crus.

### Paraesophageal laceration and lower mediastinal lymphadenectomy

**Figure 1.**
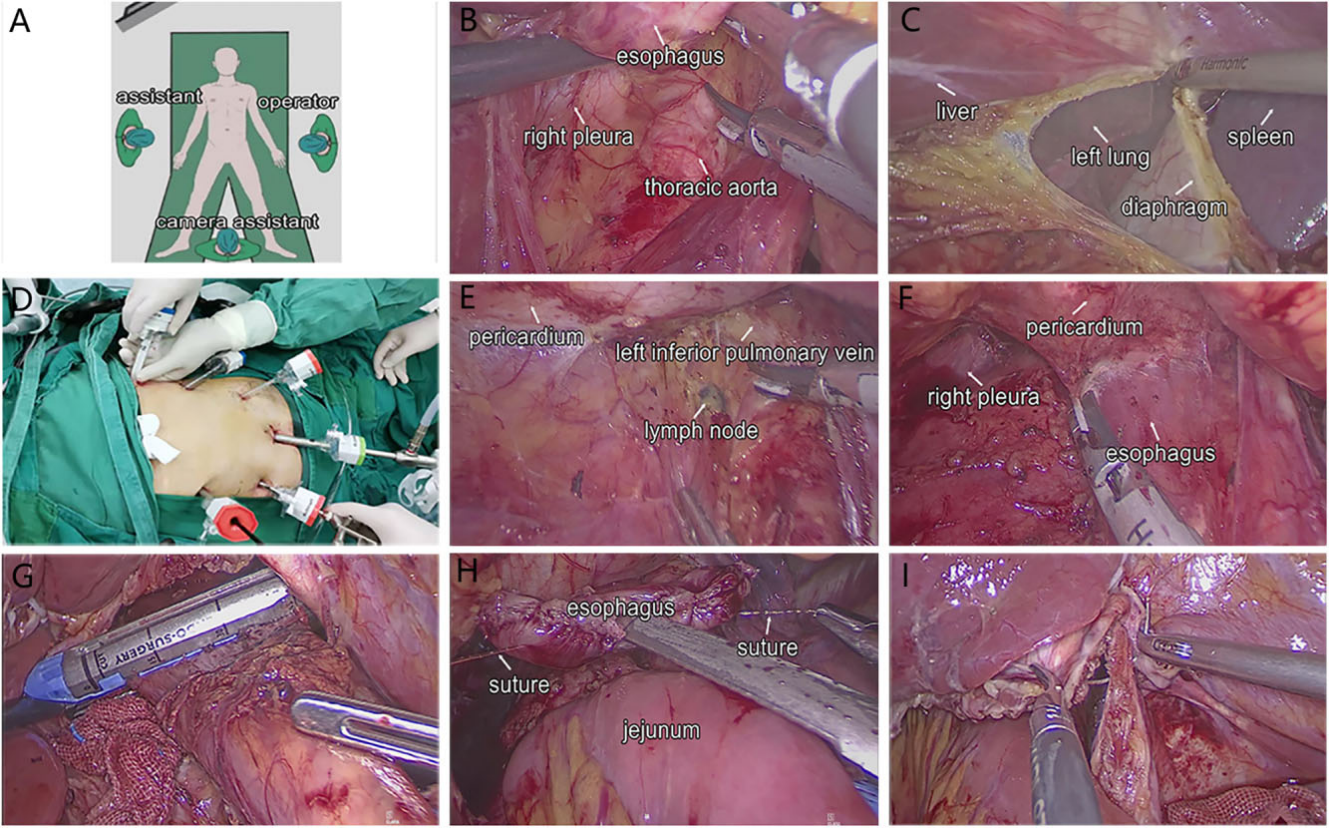
(A) Operator's position; (B) partial No. 112pulR and No. 110 LNs dissection; (C) diaphragm incision; (D) insertion of the thoracic trocar; (E) exposure of the left inferior pulmonary vein through the thoracic port; (F) denuding of the pericardium; (G) the esophagus was transected using the stapler through the thoracic port; (H) the overlap esophagojejunostomy; (I) diaphragmatic incision closure.

The operator stood as shown in Figure 1A. The phrenicoesophageal ligament surrounding the esophagus was divided to expose the esophagus. First, the fascia space on the right side of the esophagus was divided and the subcardinal sac was exposed. Then, the right pulmonary ligament LNs (No. 112pulR) and the anterior thoracic para-aortic LNs (No. 112aoA) located between the esophageal hiatus and the inferior pericardium and between the right pleura and the left edge of the thoracic aorta were dissected (Figure 1B). Second, the left diaphragm was incised ∼5–7 cm (Figure 1C) and a 12-mm port was inserted along the left anterior axillary line of the sixth-to-seventh intercostal space (Figure 1D) as the thoracic main operative port for lower mediastinal lymphadenectomy and digestive tract reconstruction. The upper diaphragm was suspended and the supradiaphragmatic LNs (No. 111) were removed through the thoracic operation port. Third, the left inferior pulmonary ligament was dissected upward to the left inferior pulmonary vein (Figure 1E) to dissect the left pulmonary ligament LNs (No. 112pulL). The No. 112aoA LNs were dissected to the level of the left inferior pulmonary vein subsequently. Fourth, the posterior pericardium was denuded (Figure 1F) and the pulmonary ligament LNs (No. 112pul) were dissected completely using the energy device through the thoracic port. Finally, the esophagus was denuded from cranial to caudal and the lower thoracic paraesophageal LNs (No. 110) were dissected after transecting the esophagus 5 cm above the tumor using the stapler through the thoracic port (Figure 1G). The specimen was extracted through a 3- to 5-cm assisted abdominal incision in the midline and the proximal esophagus margin was immediately sent for intraoperative frozen pathological examination.

### Reconstruction

The Roux-en-Y esophagojejunostomy was used during the digestive tract reconstruction. The side-to-side jejunojejunostomy and the Roux-limb were completed through the mini assisted incision. After the negative proximal margin was confirmed by the intraoperative rapid frozen section, the esophagojejunostomy was performed. Two barbed threads were sutured on the middle part of the esophageal stump 1 cm apart from each other. The overlap esophagojejunostomy was performed through the traction of the two sutures and the introduction of a gastric tube (Figure 1H). A drainage tube was inserted into the left thoracic cavity routinely and the diaphragm incision was closed using an absorbable suture to prevent a hiatus hernia (Figure 1I).

## Discussion

According to the fifth edition of the Japanese Gastric Cancer Treatment Guidelines, the TH approach is recommended for Siewert type II AEG with esophageal invasion of <3 cm [5]. However, lower mediastinal lymphadenectomy and digestive tract reconstruction via the TH approach are difficult because of the deep position of the lower mediastinum, the narrow surgical area, and obstruction by surrounding organs. The open left diaphragm approach [6] can expand the operative field and avoid the obstruction of the diaphragm. However, denuding the posterior pericardium, exposure of the left inferior pulmonary vein, and reconstruction of the digestive tract are difficult.

Our technique expanded the operative field by incising the left diaphragm and alleviating the “chopsticks effect” by increasing the thoracic port. The merits of this technique can be listed as follows: first, the expanded operative field makes the lower mediastinal lymphadenectomy and digestive tract reconstruction easier and safer; second, it can avoid changing position during the operation; third, it provides a negative proximal margin as far as possible without increasing the difficulty of digestive tract reconstruction; fourth, it can fully expose the left lower pulmonary vein.

Notably, there are some points for attention. During the dissection of the mediastinal LNs, the assistant needs to block the heart, which may lead to transient arrhythmia. If serious arrhythmia occurs during the operation, the operation can be suspended until the normal rhythm is restored. In addition, due to the involvement of thoracic and abdominal anatomy, it is recommended that the operation should be performed in an experienced center.

Ethical statement: The study was approved by the local ethics committee of Guangdong Provincial Hospital of Chinese Medicine (No. ZF2018-219).

## Funding

Project of the “double first class” and collaborative innovation team of a high-level university discipline [2021xk48].
